# Development of Shinai-Embedded IMU-Based Sensing System for Motion Analysis of Kendo Swings

**DOI:** 10.3390/s26113356

**Published:** 2026-05-26

**Authors:** Yuta Ogai, Masaomi Sanekata

**Affiliations:** Faculty of Engineering, Tokyo Polytechnic University, Atsugi, Kanagawa 243-0297, Japan; sanekata@eng.t-kougei.ac.jp

**Keywords:** inertial measurement unit (IMU), kendo, sports, wearable, motion analysis, embedded system

## Abstract

In recent years, wearable sensing technologies have been widely used for motion analysis in sports; however, in kendo, motion evaluation still largely relies on subjective assessment, and quantitative approaches remain limited. This study proposes an embedded inertial measurement unit (IMU)-based sensing system integrated into a bamboo sword (shinai) for the motion analysis of kendo swings. The system incorporates a compact IMU and a microcontroller within the shinai, enabling unobtrusive measurement under realistic training conditions without affecting usability. Using the acquired sensor data, motion-related acceleration components were extracted with orientation estimation using the error-state Kalman filter (ESKF) based on six-axis IMU data, followed by gravity compensation and feature extraction based on the peak characteristics of the swing motion. The experimental results show that experienced practitioners exhibited significantly higher peak acceleration (*p* = 0.002) and smaller peak width (*p* = 0.022) than novice practitioners, indicating sharper and more efficient motion. No significant differences were observed in the secondary peak ratio. These results demonstrate that the proposed system can quantitatively capture the motion characteristics of kendo swings and distinguish practitioners of different proficiency levels, which highlights its potential for objective motion analysis and training support in kendo.

## 1. Introduction

### 1.1. Motion Characteristics of Kendo Swings

In recent years, wearable sensing technologies have been widely utilized for motion analysis in sports. In particular, inertial measurement units (IMUs) have gained significant attention due to their compact size, low cost, and ability to measure acceleration and angular velocity. IMU-based sensing has been applied in a wide range of domains including rehabilitation, gait analysis, athlete monitoring, and sports performance evaluation [1,2,3,4,5,6,7,8,9]. In addition, quantitative motion analysis using IMUs has been actively studied for swing-related sports such as golf, ice hockey, and baseball, as well as in combat sports, for training support and performance improvement [10,11,12,13]. Recent studies have also investigated wearable sensing and biomechanical analysis in combat sports and martial arts, including striking analysis, training assessment, and motion classification using inertial sensors and related wearable technologies [14,15,16]. In contrast, in the Japanese sword fighting-based martial art kendo, motion evaluation still relies primarily on subjective judgment by instructors. Therefore, there is a need to establish methods for the objective and quantitative evaluation of kendo motion. However, the temporal structure of kendo swings has not been sufficiently analyzed using sensor data.

Recent studies on IMU-based orientation estimation have reported approaches applicable to both IMU-only and MARG-based systems (e.g., [17,18]) that enable the extraction of motion-related acceleration components through orientation estimation and gravity compensation.

**Figure 1:**
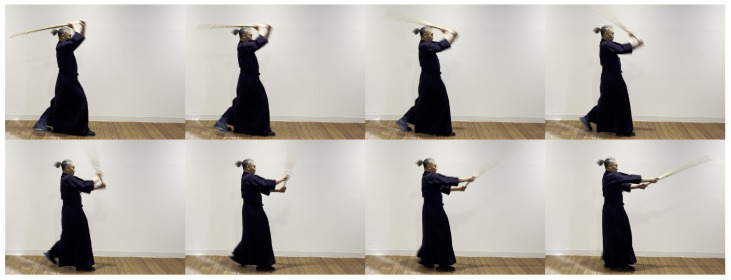
The moment of striking during a swing motion performed by an experienced practitioner. Frames are extracted from the video every 0.033 s and displayed in a grid, with the first four frames in the top row and the subsequent frames in the bottom row.

An example of a swing motion is provided in the Supplementary Material (Figure S1). As shown here, kendo swings are temporally continuous movements characterized by the periodic repetition of motions corresponding to strikes. Figure 1 shows the moment of striking during a swing motion performed by an experienced practitioner. In such movements, distinct peaks appear in the acceleration signal, corresponding to the moment of striking. In this study, these peaks are used as indicators of motion characteristics.

### 1.2. Related Work and Limitations

Various studies have applied sensors such as accelerometers, IMUs, and motion capture for kendo motion analysis. Ref. [19] investigated kendo striking motion using tri-axial accelerometers attached to multiple body segments, including the waist, forearms, and ankles, and analyzed the temporal coordination patterns of skilled and novice practitioners. They demonstrated that acceleration measurements can provide useful information for evaluating kendo skill, particularly in terms of movement timing and coordination. However, their approach relied on multiple sensors attached to the body, which may affect natural motion and requires a relatively complex measurement setup. In contrast, the present study employs a sensing system embedded directly within the shinai, enabling unobtrusive measurement without modifying the practitioner’s body or movement. Furthermore, while Ref. [19] focused on the qualitative interpretation of motion characteristics, this study aims to extract the quantitative features of swing motion under realistic training conditions by using a simplified measurement configuration.

In addition to sensor-based approaches, recent studies have investigated kendo motion using motion-capture and mixed-reality systems. Ref. [20] proposed a mixed reality (MR)-based training system for kendo in which practitioners interact with a digital human representation of themselves using a head-mounted display and haptic devices. Their work focuses on subjective experience and self-reflection through autoethnography, particularly in relation to the concept of “qi” in kendo. Ref. [21] analyzed men-strike motion in skilled practitioners (eighth-dan) using a VICON-based motion-capture system and reported commonalities and individual variability in joint and shinai angle time-series patterns, providing detailed biomechanical insights into high-level kendo technique. In contrast to these approaches, the present study focuses on practical and unobtrusive quantitative motion analysis using an embedded IMU-based sensing system applicable in routine training environments.

Refs. [22,23] proposed IMU-based systems for recognizing kendo strike activities using multiple sensors attached to both the body and the shinai. Their approaches focused on activity recognition and classification, employing machine learning and time-series analysis techniques to identify discrete action types such as Men, Kote, and Do. In contrast, the present study focuses on analyzing the motion characteristics of kendo swing dynamics rather than classifying action types. In particular, this study aims to quantify differences between experienced and novice practitioners based on extracted motion features and statistical analysis. Furthermore, while previous studies have primarily addressed activity recognition tasks, the present work emphasizes the evaluation of skill-related motion characteristics under realistic training conditions. These differences in research objectives and analysis approaches indicate that the studies are complementary rather than directly comparable.

Ref. [24] investigated grip pressure during kendo attacks by instrumenting a shinai with pressure sensors and analyzing the distribution of forces between the left and right hands across different phases of the attack, providing valuable insights into hand usage and attack phases in kendo. However, their approach focused on force measurement rather than motion analysis and did not capture the kinematic characteristics of the swing motion itself. In contrast, the present study utilizes IMU-based sensing to analyze acceleration signals associated with swing dynamics, enabling the extraction of motion-related features. These differences in sensing modality and analysis objectives do not enable a direct comparison but highlight complementary aspects of kendo performance analysis.

In addition, many IMU-based methods for attitude estimation use magnetometers, which can be susceptible to magnetic field disturbances in indoor environments and during highly dynamic movements, making stable measurement difficult. Moreover, research on sensor-based measurement in kendo is limited compared with that in other sports, and there has been limited investigation of measurement methods applicable to real training environments. The embedding of sensing systems within a shinai has not been sufficiently investigated in the context of motion analysis. Our previous work proposed a basic IMU-based measurement system embedded in a shinai [25]; however, it did not address magnetometer-free orientation estimation or detailed quantitative analysis of motion characteristics.

### 1.3. Purpose and Contribution

In this study, we propose a motion measurement method that embeds an IMU and measurement system inside the shinai. The system enables unobtrusive measurement in a natural state by integrating sensors, microcontrollers, and batteries inside the shinai. In addition, this study applies the error-state Kalman filter (ESKF) for attitude estimation and gravity compensation by using only six-axis IMU data consisting of acceleration and angular velocity, without using a magnetic sensor. This approach aims to stably extract motion-induced acceleration components even under highly dynamic conditions.

The objective of this study is to quantitatively capture the characteristics of kendo motion by using the proposed system and to clarify the differences in motion between experienced and novice practitioners.

### 1.4. Paper Structure

The structure of this paper is as follows: In Section 2, the configuration of the proposed system and the motion analysis method are described. In Section 3, the experimental results are presented. In Section 4, the results are discussed. Finally, in Section 5, this study is summarized.

## 2. Materials and Methods

### 2.1. System Overview

**Figure 2:**
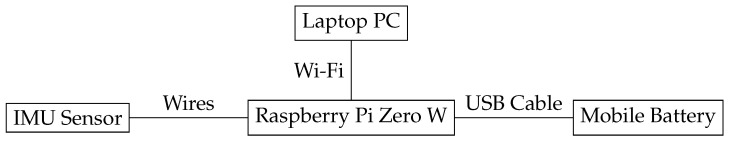
Overview of the proposed embedded IMU-based sensing system for kendo swing analysis.

The proposed sensing system is designed to measure kendo swing motion in a natural training environment without interfering with user performance. Figure 2 shows an overview of the proposed sensing system. The system consists of an IMU sensor, a battery, and a single-board computer (Raspberry Pi Zero W, Raspbian GNU/Linux 11 (Bullseye), Cambridge, UK) embedded inside the shinai, as well as an external PC. The Raspberry Pi Zero W processes data from the sensor and transmits them to the external PC via Wi-Fi. The acquired IMU data are processed through a series of steps including resampling, orientation estimation, gravity compensation, and feature extraction.

### 2.2. Hardware Configuration

The sensor used is an IMU, specifically a module based on the MPU-9250 (InvenSense Inc., San Jose, CA, USA), a nine-axis sensor that integrates a tri-axial accelerometer, a tri-axial gyroscope, and a tri-axial magnetometer; however, the magnetometer was not used in this study to reduce computational and data-acquisition load and to avoid possible magnetic disturbances in indoor environments [1,26]. The accelerometer measurement range was set to ±16g, and the gyroscope range was set to ±1000dps. Prior to data collection, we performed a basic static verification of the IMU sensor, placing it in known orientations and confirming that the measured acceleration corresponded to approximately 1 g along the expected axis, ensuring the consistency of the sensor output. The circuit diagram combining the Raspberry Pi Zero W and the MPU-9250 is shown in the Supplementary Material (Figure S2).

We used a lithium-ion mobile battery, DE-M04L-3200BK from Elecom Co., Ltd., Osaka, Japan (output: 5 V −2.1 A, 3200 mAh; diameter of 2.4 cm, length of 10.1 cm, and weight of 72 g), connected to the Raspberry Pi Zero W via a USB cable for power supply. Its compact size allows it to be mostly housed inside the handle of the shinai.

We used a typical shinai with a total length of approximately 120 cm and an original weight of 513 g. The center of mass was located approximately 62 cm from the tip. To accommodate the components, the bamboo was partially hollowed out, resulting in a reduction in the shinai’s weight from 513 g to 471 g.

The Raspberry Pi Zero W was configured to support Python 3.9.2 and an SSH server. During sensor data acquisition, an external device connected via SSH to execute a Python script that retrieved the data. The experiments were conducted in kendo training halls, so a small Wi-Fi router was used to connect the Raspberry Pi Zero W and the external PC.

### 2.3. Embedded Implementation

**Figure 3:**
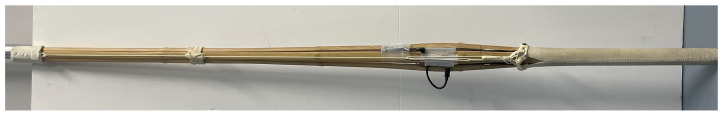
Embedded implementation of all components inside the handle of the bamboo sword (shinai).

Figure 3 shows the embedded implementation of the proposed sensing system inside the handle of the shinai. After embedding the components, the weight of the shinai became approximately 557 g (44 g heavier than the original shinai), and the center of mass shifted to approximately 6 cm toward the handle.

**Figure 4:**
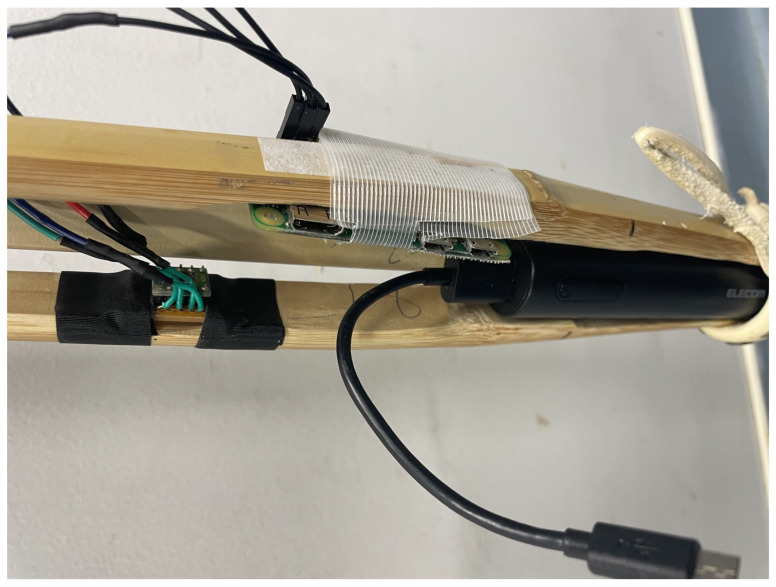
The internal structure of the embedded system as revealed by slightly opening the shinai.

Figure 4 shows the internal structure of the embedded system as revealed by slightly opening the shinai. The IMU sensor and the Raspberry Pi Zero W were fixed to the bamboo of the shinai by using tape. The orientation of the IMU accelerometer was defined such that the positive Z-axis pointed in the direction of raising the shinai, the positive X-axis pointed towards the tip of the shinai, and the positive Y-axis pointed to the left side of the shinai (from the perspective of the practitioner).

### 2.4. Data Acquisition Procedure

The sampling frequency was set to 20 Hz, and the measured data were recorded in comma-separated values (CSV) format. At the beginning of the program, the memory space necessary for saving data during operation was allocated. The program includes a process that waits until a specified length of time (0.05 s for 20 Hz) has elapsed before acquiring the next data point. The sampling frequency was set to 20 Hz because higher frequencies may cause the data acquisition process to lag behind. This frequency is the upper limit of the sampling frequency that the system can handle without lagging in data processing. Even with the 20 Hz setting, there may be some variability in the actual data acquisition time, so linear interpolation is applied to create a strictly 20 Hz evenly spaced dataset for analysis.

In the experiment, participants performed vertical swings (suburi) for approximately 10 s, during which data were recorded. Participants were divided into two groups: experienced practitioners and novice practitioners. The experienced group consisted of 10 males in their 20 s, 1 male in his 40 s, 1 male in his 70 s, and 3 females in their 20 s. Among the experienced practitioners, two females had 2 years of kendo experience, while all other experienced practitioners had 9 or more years of kendo experience. The novice group consisted of individuals in their 20 s with little to no experience in kendo. The participants in the experiment included 24 individuals, consisting of 19 males (12 experienced and 7 novice) and 5 females (3 experienced and 2 novice). Two trials were recorded for each participant, where the second trial was used for analysis in order to reduce variability associated with initial familiarization with the measurement setup. When the second trial was unavailable due to sensor malfunction, the first trial was used instead. Preliminary analysis confirmed that the main results were consistent regardless of trial selection, indicating that the findings are robust to this choice. A questionnaire assessing the effects of integrating the system was also administered to the 15 experienced practitioners; the participants responded to each question using a 5-point Likert scale: “Strongly Agree,” “Agree,” “Neutral,” “Disagree,” and “Strongly Disagree.”

### 2.5. Orientation Estimation

Quaternion-based orientation estimation methods using IMU data have been extensively studied for motion tracking, robotics, and wearable sensing applications [26]. Representative approaches include complementary filters, nonlinear observers, Extended Kalman Filters (EKFs), and Error-State Kalman Filters (ESKFs) [17,27,28,29,30]. These methods enable stable estimation of orientation and gravity compensation even under dynamic motion conditions. Many studies have also reported on improving the accuracy of orientation estimation by integrating IMU sensors [31]. In this study, unlike the method by Wei et al., no correction using magnetometer data is performed, and orientation estimation is conducted using only gyroscope and accelerometer data, given its sensitivity to magnetic disturbances, particularly in indoor environments and highly dynamic motion conditions such as kendo swings [1,26]. Compared with methods such as the EKF, the ESKF showed less drift in orientation estimation for the types of data collected in this study. The gravity component is removed from the acceleration data by using the estimated orientation to calculate the acceleration due to motion.

The ESKF state consists of the nominal orientation quaternion (*q*) and gyroscope bias ($b_{g}$). The error state (δx) is defined as

(1) $$\begin{array}{c} \delta x=\left[\begin{array}{c} \delta \theta \\ \delta b_{g} \end{array}\right], \end{array}$$

where δθ represents the small attitude error, and $\delta b_{g}$ represents the gyroscope bias error.

In the prediction step, the nominal quaternion $q_{k}$ is propagated using the bias-corrected angular velocity:

(2) $$\begin{array}{c} \omega_{k}=\omega_{m,k}-b_{g,k}, \end{array}$$

(3) $$\begin{array}{c} q_{k+1}=q_{k}+\frac{1}{2}q_{k}⊗\omega_{k}\Delta t, \end{array}$$

where $\omega_{m,k}$ denotes the measured angular velocity, $\omega_{k}$ represents the bias-corrected angular velocity used for state propagation, the subscript *k* denotes the discrete time step, Δt is the sampling interval, and the operator ⊗ denotes quaternion multiplication.

The error-state transition matrix ($F_{k}$) is obtained by performing first-order linearization of the continuous-time error dynamics, resulting in the approximation

(4) $$\begin{array}{c} F_{k}=\left[\begin{array}{cc} I-{\left[\omega_{k}\right]}_{\times }\Delta t & -I\Delta t\\ 0 & I \end{array}\right], \end{array}$$

where ${\left[\omega_{k}\right]}_{\times }$ is the skew-symmetric matrix of $\omega_{k}$, and *I* is the identity matrix.

The error-state covariance matrix ($P_{k}$) is propagated as

(5) $$\begin{array}{c} P_{k+1}=F_{k}P_{k}F_{k}^{T}+Q_{k}, \end{array}$$

where $Q_{k}$ is the process noise covariance matrix. In this implementation, the process noise covariance was set as

(6) $$\begin{array}{c} Q_{k}=\left[\begin{array}{cc} \sigma_{\theta }^{2}I & 0\\ 0 & \sigma_{b}^{2}I \end{array}\right], \end{array}$$

where $\sigma_{\theta }=0.02$ and $\sigma_{b}=0.002$ were used for the attitude-error and gyroscope-bias components, respectively.

To update the accelerometer, only the direction of the measured acceleration $z_{k}$ is used:

(7) $$\begin{array}{c} z_{k}=\frac{a_{m,k}}{|a_{m,k}|}, \end{array}$$

where $a_{m,k}$ denotes the measured acceleration vector at time step *k*.

The predicted gravity direction $h(q_{k})$ in the sensor frame is given by

(8) $$\begin{array}{c} h\left(q_{k}\right)=q_{k}^{*}⊗g_{u}⊗q_{k}, \end{array}$$

where $g_{u}={\left[0,0,1\right]}^{T}$ is the unit gravity vector in the world frame, treated as a pure quaternion (i.e., with zero scalar part) when used in quaternion multiplication.

The innovation ($y_{k}$) is defined as

(9) $$\begin{array}{c} y_{k}=z_{k}-h\left(q_{k}\right). \end{array}$$

Jacobian $H_{k}$ with respect to the error state is given by

(10) $$\begin{array}{c} H_{k}=\left[\begin{array}{cc} {\left[h\left(q_{k}\right)\right]}_{\times } & 0 \end{array}\right], \end{array}$$

where ${\left[h\left(q_{k}\right)\right]}_{\times }$ is the skew-symmetric matrix of the predicted gravity direction.

The measurement noise covariance ($R_{k}$) is adaptively scaled according to the deviation of the acceleration magnitude from the gravity value:

(11) $$\begin{array}{c} r_{k}=\frac{\left||a_{m,k}|-g\right|}{g}, \end{array}$$

(12) $$\begin{array}{c} R_{k}=\sigma^{2}\left(1+{\left(\frac{r_{k}}{0.2}\right)}^{2}\right)I, \end{array}$$

where $r_{k}$ represents the relative deviation from the gravity magnitude, and $g={\left[0,0,9.80665\right]}^{T}$ is the gravity vector expressed in the world frame. The nominal measurement noise standard deviation is set to σ=0.05.

The Kalman gain ($K_{k}$) is computed as

(13) $$\begin{array}{c} K_{k}=P_{k}H_{k}^{T}{\left(H_{k}P_{k}H_{k}^{T}+R_{k}\right)}^{-1}, \end{array}$$

where $P_{k}$ is the error-state covariance matrix, and $R_{k}$ is the measurement noise covariance matrix.

After correction, the covariance matrix is updated using the Joseph form:

(14) $$\begin{array}{c} P_{k}\leftarrow \left(I-K_{k}H_{k}\right)P_{k}{\left(I-K_{k}H_{k}\right)}^{T}+K_{k}R_{k}K_{k}^{T}. \end{array}$$

The error state $\delta x_{k}$ is estimated as

(15) $$\begin{array}{c} \delta x_{k}=K_{k}y_{k}. \end{array}$$

The nominal state ($q_{k}$) is then corrected using the estimated error:

(16) $$\begin{array}{c} q_{k}\leftarrow q_{k}⊗\delta q,\;\delta q\approx \left[\begin{array}{c} 1\\ \frac{1}{2}\delta \theta \end{array}\right], \end{array}$$

(17) $$\begin{array}{c} b_{g}\leftarrow b_{g}+\delta b_{g}. \end{array}$$

Finally, the gravity-compensated linear acceleration ($a_{\mathrm{lin},k}$) at time step *k* is obtained as

(18) $$\begin{array}{c} a_{\mathrm{lin},k}=a_{m,k}-R{\left(q_{k}\right)}^{T}g. \end{array}$$

Absolute yaw orientation cannot be uniquely determined from 6-axis IMU data alone. However, yaw rotation does not affect the direction of gravity and therefore has limited influence on gravity compensation. In this study, the estimated orientation is primarily used to remove the gravity component from acceleration signals; thus, the accurate estimation of roll and pitch is sufficient for the subsequent motion analysis.

### 2.6. Feature Extraction

The overall signal-processing pipeline consists of the following steps: (1) acquisition of IMU data, (2) resampling to a uniform 20 Hz using linear interpolation, (3) orientation estimation using the ESKF, (4) gravity compensation, (5) the smoothing of the acceleration norm, (6) motion onset detection, (7) peak detection, and (8) feature extraction.

Motion onset detection and peak detection are performed based on the gravity-compensated acceleration data. The norm of the tri-axial acceleration vector is calculated at each time point and smoothed using a moving average filter with a window size of 3 samples to reduce noise. The norm serves as an indicator of motion intensity that is independent of direction and is used for motion onset detection. Specifically, after excluding the initial 0.5 s as a warm-up period to avoid transient effects, a robust threshold was defined as the median plus 1.5 times the median absolute deviation (MAD) of the acceleration norm, and motion onset was defined as the point where this threshold was exceeded for at least 3 consecutive samples.

Peak detection based on local maxima and thresholding is commonly used in time-series analysis and inertial-sensor signal processing [32]. Z-axis acceleration was used for the detection of individual strikes. Main peaks were extracted from the Z-axis acceleration signal as local maxima exceeding a saliency threshold defined as max (noise scale, 1.0 m/s^2^). A local maximum was defined as a point whose value is greater than its immediately adjacent samples. The noise scale was computed as 1.4826 times the median absolute deviation (MAD) of the Z-axis acceleration signal, providing an estimate of the standard deviation under the assumption of a normal distribution. The MAD-based scaling factor (1.4826), widely used in robust statistics, provides a robust estimate of the standard deviation under Gaussian assumptions [33]. A minimum time interval of 0.7 s between peaks was imposed to suppress false detection instances. Moreover, the first and last peaks were excluded to eliminate the influence of the initial and final transient phases.

Since a small secondary peak following the main peak appeared to be characteristic in the acceleration waveform of experienced practitioners, secondary peaks were also extracted. These were defined as local maxima occurring after the main peak within the same swing cycle. To avoid spurious detection instances, we considered only peaks exceeding 15% of the corresponding main peak amplitude and located within a time window excluding 0.1 s immediately after the main peak and 0.1 s before the next peak. It should be noted that secondary peaks are defined relative to each detected main peak within a swing cycle and are not reclassified as main peaks, even if their amplitude is relatively large.

The complete signal-processing algorithm is summarized in Listing 1 in Python-like pseudocode.

**Listing 1.** Signal-processing algorithm (Python-like pseudocode).

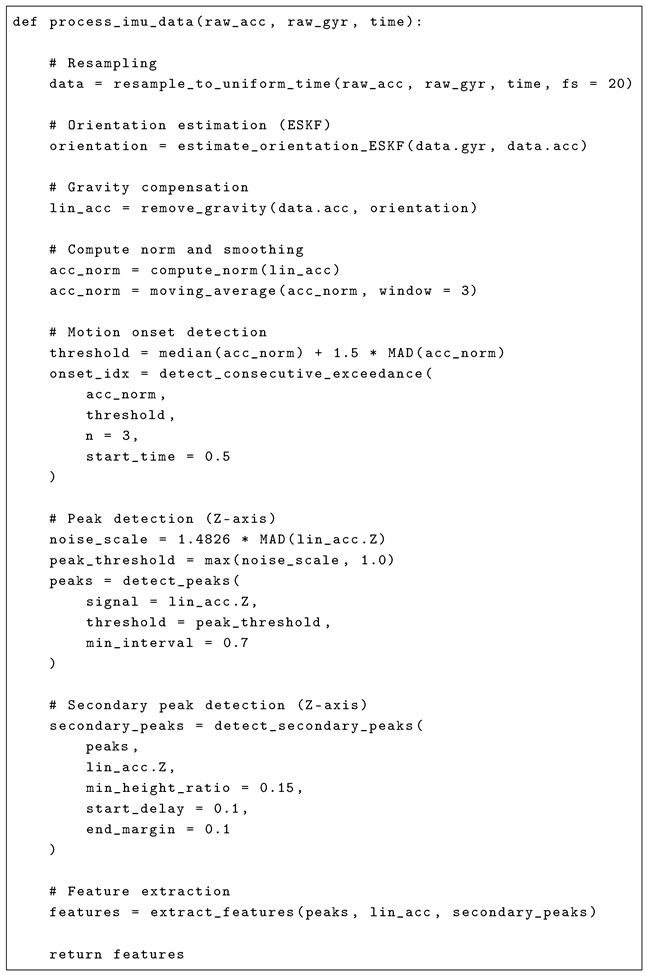



Statistical analysis was performed using Welch’s *t*-test for group comparisons. Welch’s *t*-test, which is robust to unequal variances and sample sizes between groups, was adopted [34]. In addition, normality was assessed using the Shapiro–Wilk test, and the Mann–Whitney U test was performed as a non-parametric alternative. The significance level for all statistical tests was set to 0.05.

## 3. Results

### 3.1. Overview of Measured Signals

The proposed sensing system successfully recorded inertial data during kendo swing motion for all participants. The recorded signals included tri-axial acceleration and angular velocity, which were further processed to obtain gravity-compensated acceleration. As raw acceleration data contain gravity components and may exhibit drift, making it difficult to accurately represent motion characteristics, the gyroscope data and the ESKF were used to perform orientation estimation and obtain gravity-compensated acceleration data. Examples of raw acceleration and gyroscope signals are shown in the Supplementary Material (Figures S3 and S4).

**Figure 5:**
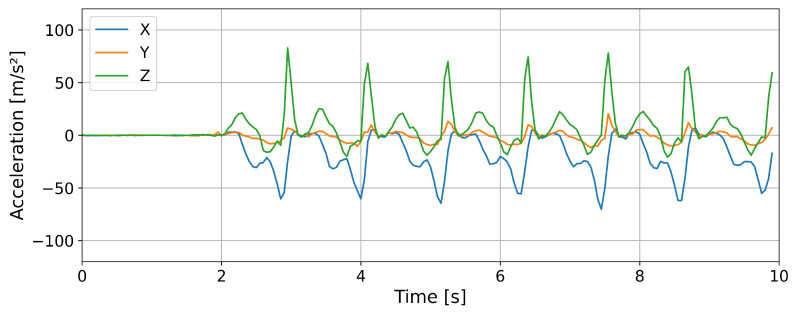
Gravity-compensated acceleration measured from experienced kendo practitioner A during a swing trial, corrected with the ESKF.

**Figure 6:**
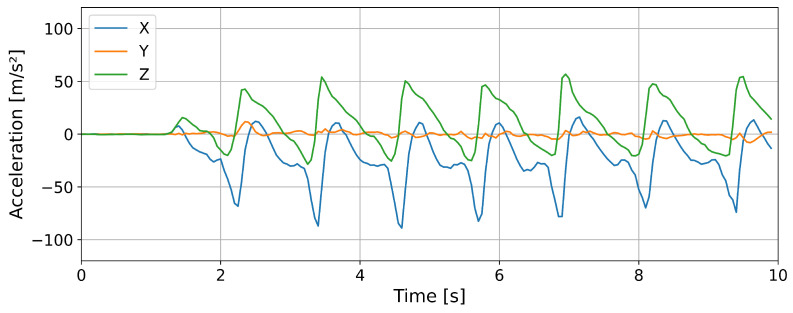
Example of gravity-compensated acceleration measured from novice kendo practitioner P during a swing trial, corrected with the ESKF.

Figure 5 and Figure 6 show examples of gravity-compensated acceleration for experienced practitioner A and novice practitioner P, respectively. During the vertical swing motion, it can be observed that experienced practitioners exhibit sharper peaks in the Z-axis direction compared with novice practitioners.

**Figure 7:**
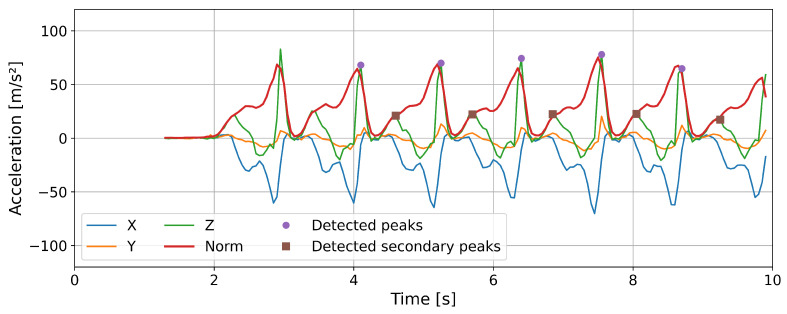
Example of gravity-compensated acceleration signals and detected peaks. The main peaks and second peaks in the Z-axis signal clearly correspond to each swing cycle. The first and last peaks are excluded to eliminate the influence of the initial and final transient phases.

Figure 7 shows the automatically extracted peaks of the Z-axis of the processed acceleration data from an experienced kendo practitioner. The visually identifiable peaks in the Z-axis direction correspond well with the automatically calculated peaks of the Z-axis.

### 3.2. Questionnaire Results

Among the 15 experienced practitioners, to the question “Did you feel that the shinai used in this experiment was heavier than a normal shinai?”, 9 responded “Strongly Disagree”, and 6 responded “Disagree.” To the question “Did you feel that the shinai used in this experiment was more difficult to swing than a normal shinai?”, 9 responded “Strongly Disagree”, and 6 responded “Disagree.”

### 3.3. Comparison of Main Peak Acceleration

The main peak acceleration in the Z-axis direction for all detected swings was obtained for each trial of each participant, and the mean and standard deviation were calculated. A comparison of the experienced and novice groups was then performed.

**Figure 8:**
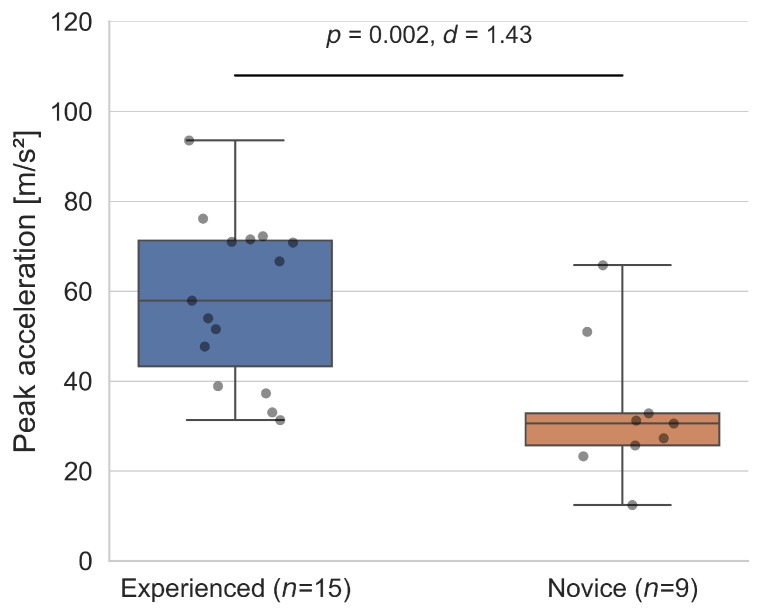
Comparison of mean main peak acceleration of experienced and novice practitioners. Boxes represent medians and interquartile ranges, whiskers indicate the full data range, and individual data points are overlaid. The experienced group showed significantly higher values than the novice group (Welch’s *t*-test, *p* = 0.002, Cohen’s d = 1.43).

The results are shown in Figure 8. The experienced group showed significantly higher main peak acceleration values than the novice group (Welch’s *t*-test, *p* = 0.002), with a large effect size (Cohen’s d = 1.43). Normality was assessed using the Shapiro–Wilk test (experienced: *p* = 0.473; novice: *p* = 0.178), indicating no significant deviation from normality. The Mann–Whitney U test also indicated a significant difference between the groups (*p* = 0.002), consistent with the results of Welch’s *t*-test.

### 3.4. Comparison of Main Peak Full Width at Half Maximum (FWHM)

**Figure 9:**
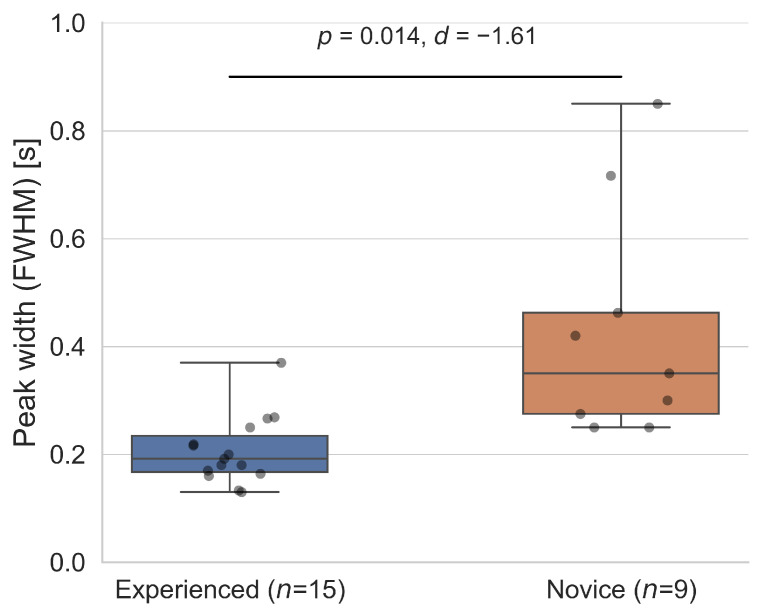
Comparison of peak width (FWHM) of experienced and novice practitioners. Boxes represent medians and interquartile ranges, whiskers indicate the full data range, and individual data points are overlaid. The novice group showed significantly larger peak width than the experienced group (Welch’s *t*-test, *p* = 0.014, Cohen’s d = −1.61).

The Full Width at Half Maximum (FWHM) of the main peak in the Z-axis direction for all detected swings was obtained for each trial of each participant, and the results of the comparison of the experienced and novice groups are shown in Figure 9. The novice group showed a significantly larger peak width (FWHM) than the experienced group (Welch’s *t*-test, *p* = 0.014), with a large effect size (Cohen’s d = −1.61). Normality was assessed using the Shapiro–Wilk test (experienced: *p* = 0.102; novice: *p* = 0.037), indicating that normality may not be fully satisfied. The Mann–Whitney U test also confirmed a significant difference between the groups (*p* = 0.001), consistent with the results of Welch’s *t*-test.

### 3.5. Comparison of Secondary Peak Ratio

For each practitioner, the ratio of the main peak to the secondary peak, referred to as the secondary peak ratio, was calculated and compared between the experienced and novice groups. One experienced practitioner and two novice practitioners were excluded from this analysis because the secondary peak could not be detected for them.

**Figure 10:**
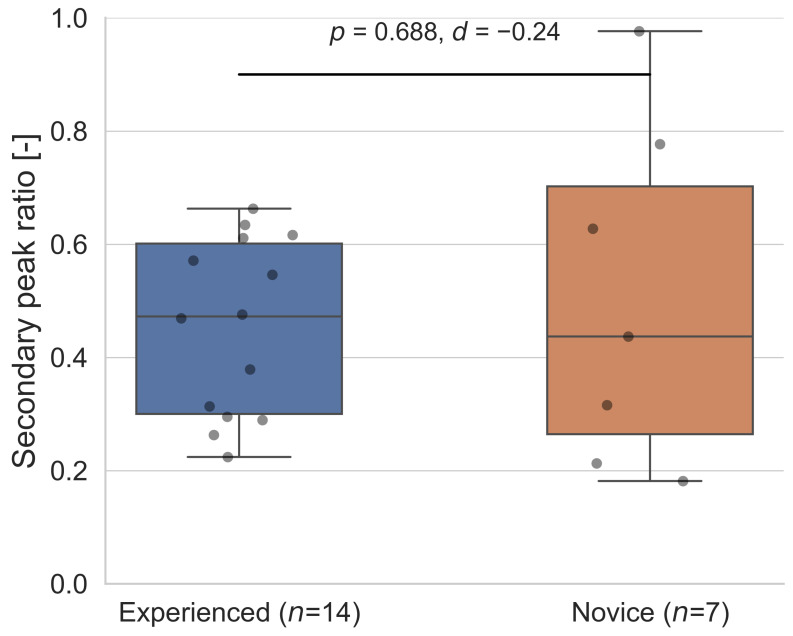
Comparison of secondary peak ratio of experienced and novice practitioners. Boxes represent medians and interquartile ranges, whiskers indicate the full data range, and individual data points are overlaid. One experienced practitioner and two novice practitioners were excluded because secondary peaks could not be reliably detected. No significant differences were observed between the groups (Welch’s *t*-test, *p* = 0.688, Cohen’s d = −0.24). However, the novice data points appear to form two clusters, suggesting potential subgroups among the novice practitioners.

The results are shown in Figure 10 as box plots. No significant differences were observed in the secondary peak ratio between the experienced and novice groups (Welch’s *t*-test, *p* = 0.688), with a small effect size (Cohen’s d = −0.24). Normality was assessed using the Shapiro–Wilk test (experienced: *p* = 0.127; novice: *p* = 0.563), which indicated no significant deviation from normality. The Mann–Whitney U test also showed no significant differences between the groups (*p* = 0.913), consistent with the result of Welch’s *t*-test. However, the novice data points appear to form two clusters, suggesting potential subgroups among the novice practitioners.

## 4. Discussion

### 4.1. Effectiveness of the Proposed System

By embedding the sensor and measurement system within the shinai, this study achieved unobtrusive measurement of motion. The questionnaire results also confirmed that the participants did not feel any discomfort regarding weight or swingability, suggesting that the system is applicable in real training environments. The measured data enabled the quantitative analysis of the motion characteristics of experienced and novice practitioners, indicating that the data quality is sufficient for reliable motion analysis. The sampling frequency was limited to 20 Hz due to the processing limitations of the Raspberry Pi Zero W. While this frequency is insufficient to capture high-frequency dynamics associated with the impact phase of kendo strikes, the focus of this study is on the overall swing motion rather than the detailed impact process. The swing motion analyzed in this study has a typical cycle duration of approximately 0.5–1.0 s, corresponding to dominant frequency components below approximately 2 Hz. In addition, the peak width (FWHM) observed in the acceleration signal is in the order of 0.1–0.3 s, corresponding to frequency components below approximately 5–10 Hz. Therefore, the selected sampling frequency satisfies the Nyquist criterion for the motion features analyzed in this study. While high-frequency components during impact may not be captured and may introduce some degree of aliasing, their contribution to the overall signal is temporally limited, and their influence on the extracted low-frequency features is considered to be small. Accordingly, the current system is suitable for analyzing the temporal structure and characteristic features of repeated swing motions but is not intended for a detailed analysis of impact dynamics.

Although direct quantitative comparison with previous studies is not appropriate due to differences in experimental conditions, the results of this study can be interpreted in relation to existing approaches. Previous IMU-based studies have primarily focused on activity recognition or classification using multiple sensors, whereas the present study demonstrates that meaningful motion characteristics can be extracted from a simplified sensing configuration using an embedded IMU system. In particular, the ability to distinguish between experienced and novice practitioners using only an embedded sensor within the shinai suggests that the proposed approach captures essential aspects of kendo motion. This highlights the practical advantage of the system in terms of applicability to real training environments. Unlike multi-sensor systems used in previous studies, the proposed system achieves meaningful analysis with a minimal configuration, which may improve usability in practical settings. This suggests that the proposed system achieves a practical balance between simplicity and effectiveness compared with existing approaches.

The IMU sensor output was verified using a basic static calibration procedure which confirmed that the measured acceleration corresponded to approximately 1 g in known orientations. Although more rigorous calibration methods were not applied, the use of a consistent sensor configuration and an identical processing pipeline across all participants ensures that relative comparisons remain valid for the purpose of this study.

### 4.2. Effectiveness of Orientation Estimation Using 6-Axis IMU and ESKF

A key technical aspect of this study is the use of only six-axis IMU data (acceleration and angular velocity) without magnetometer data, to achieve orientation estimation and gravity compensation using the ESKF. Owing to the robustness against environmental magnetic disturbances and the processing constraints of the Raspberry Pi Zero W, this study implemented a program that does not store magnetometer data. We performed gravity compensation using the orientation estimated in post-processing, and the results suggest that the acceleration components attributable to motion can be reasonably extracted. Moreover, for the data collected in this study, the ESKF showed less drift in orientation estimation than the EKF, suggesting its suitability for highly dynamic kendo swing motion.

Although absolute yaw orientation cannot be determined without a magnetometer, its influence on the present analysis is limited. In the vertical swing motion (suburi) examined in this study, the dominant movement occurs primarily in the pitch direction, while the yaw component is relatively small. Therefore, even if yaw is not accurately estimated, its effect on gravity compensation and the extracted acceleration features is minimal. The results indicate that this approach is sufficient to capture meaningful differences between experienced and novice practitioners.

### 4.3. Interpretation of Motion Characteristics

As shown in Section 3.3, the acceleration peaks observed in experienced practitioners were higher than those in novices. This may indicate that experienced practitioners can perform more efficient movements and generate greater acceleration. Furthermore, as shown in Section 3.4, the peak width of experienced practitioners was smaller than that of novices. This may indicate that experienced practitioners can apply force momentarily and perform more efficient movements without unnecessary motion. As mentioned in Section 3.5, although no significant differences were observed in the secondary peak ratio, there may be a cluster structure in the novice data, which indicates the possibility of subgroups among novices, with some learning faster than others.

According to practical knowledge shared by a high-ranking practitioner, kendo swings involve not only a downward motion but also a forward-directed component. In addition, an instructional book states that the arms should be fully extended at the moment of the swing [35]. These descriptions suggest that skilled swings may involve coordinated motion components other than simple downward acceleration, which could contribute to the secondary peaks observed before and after the main peak. By further analyzing actual swing movements and comparing them with the measured data, it may be possible to gain a more detailed understanding of the characteristics of experienced practitioners’ movements.

### 4.4. Future Perspectives

The objective and quantitative evaluation of swing motion, which has traditionally relied on subjective assessment, is expected to enable more effective training support. For example, efficient training support can be realized by measuring peak acceleration and motion sharpness with this system and providing real-time feedback through audio or graphical displays. In addition, further analyzing the behavior of secondary peaks and the detailed waveform structure may enable the application of this system for skill stratification and the qualitative evaluation of form. Since the number of experimental participants is still small and the existence of subgroups among novices is also suggested, it is desirable to collect data from more participants in the future. There may also be differences in motion characteristics among experienced practitioners, such as university kendo club members and high-ranking instructors, so more detailed analysis within the experienced group is also a worthwhile option for future work.

The current sampling frequency of 20 Hz limits the ability to capture high-frequency dynamics associated with the impact phase of kendo strikes, which occur in the order of tens of milliseconds. However, the focus of this study is on the overall swing pattern and temporal structure rather than the detailed impact process. Therefore, the extracted features primarily reflect lower-frequency motion components. Improving the system’s performance, such as increasing the sampling frequency and extending data storage time, should be pursued in future work. It is expected that higher sampling rates can be achieved by incorporating a higher-performance processing module while maintaining the weight and balance of the shinai, enabling a more detailed analysis of rapid motion components. Currently, it is necessary to open the shinai and access the internal module for charging, so design improvements to enable wireless charging and external access would also be valuable.

While the current design of the shinai raises concerns about device damage during strikes, it is not intended for use in actual sparring. However, it is desirable to enhance shock resistance in the future to enable such use.

The method using a six-axis IMU and the ESKF demonstrated in this study can also be applied to other sports and wearable motion-analysis applications and is expected to contribute to the realization of simple and high-precision wearable measurement systems. While the present study focuses on demonstrating the effectiveness of the integrated system, a systematic ablation study to isolate the contribution of each component (e.g., orientation estimation method, gravity compensation, and peak extraction strategy) would provide further insight into the role of each design choice. Such analysis is left for future work.

## 5. Conclusions

In this study, a measurement system with an IMU embedded in a shinai was developed for the quantitative analysis of kendo swing motion. The proposed system demonstrated the feasibility of achieving orientation estimation and gravity compensation by using six-axis IMU data and applying the ESKF without relying on magnetometer data, enabling the stable extraction of acceleration components due to motion. The experimental results confirmed that experienced practitioners had significantly higher main peak acceleration and smaller peak width than novices, revealing sharper and more efficient motion characteristics. On the other hand, no significant differences were observed in the secondary peak ratio, but a cluster structure in the novice group is hypothesized. Based on the above, it is determined that the proposed method is effective for extracting the key features of kendo motion and identifying the proficiency level of practitioners, and it is anticipated to be valuable for application in training support and proficiency evaluation.

## Data Availability

The data presented in this study are available from the corresponding author upon reasonable request. The data are not publicly available due to privacy and ethical considerations.

## Supplementary Materials

The following supporting information can be downloaded at https://www.mdpi.com/article/10.3390/s26113356/s1. Figure S1: Example of a kendo swing motion performed by an experienced practitioner. Frames are extracted from the video every 0.033 s and displayed in a grid, with the first four frames in the top row and the subsequent frames in the bottom row; Figure S2: Circuit diagram combining the Raspberry Pi Zero W and the MPU-9250; Figure S3: Time series of acceleration data measured from experienced kendo practitioner A during a swing trial; Figure S4: Time series of gyroscope data measured from experienced kendo practitioner A during a swing trial; Table S1: Mean and standard deviation of peak acceleration for each trial; Table S2: Mean and standard deviation of FWHM and Secondary Peak Ratio for each trial.
