# Supplementary material for: Development of Shinai-Embedded IMU-Based Sensing System for Motion Analysis of Kendo Swings
Source: Sensors (Basel). 2026 May 26;26(11):3356. doi: 10.3390/s26113356 (PMC13259464; doi:10.3390/s26113356)
Supplement: Supplementary file 1 [file sensors-26-03356-s001.zip › sensors-4275919-supplementary.pdf]

Supplementary Material: Development of Shinai-Embedded IMU-Based Sensing System for Motion Analysis of Kendo Swings

Yuta Ogai [1] and Masaomi Sanekata [2]

[1] Tokyo Polytechnic University; ogai@eng.t-kougei.ac.jp
[2] Tokyo Polytechnic University; sanekata@eng.t-kougei.ac.jp
* Correspondence: ogai@eng.t-kougei.ac.jp

S1. Summary of Trial Data

Table S1 and Table S2 present the mean and standard deviation of peak acceleration (Mean Peak and SD Peak), FWHM (Mean FWHM and SD FWHM), and secondary peak ratio (Mean 2nd Ratio and SD 2nd Ratio) for each trial of 24 experimental participants. Participants ID A to L are male kendo practitioners, M to O are female practitioners, P to V are male novices, and W to X are female novices. Each participant performed two trials. In this study, the second trial was used for analysis in order to reduce the variability associated with initial familiarization with the measurement setup. The first trial was used only when the second trial was unavailable due to sensor malfunction (participant R). The "Selected" column indicates the trial used for statistical analysis according to the predefined rule described above. NaN values indicate cases where the secondary peak could not be reliably detected and thus the corresponding feature could not be computed.

Table S1. Mean and standard deviation of peak acceleration for each trial.

ID	Trial	Selected	Mean Peak (m/s^2)	SD Peak (m/s^2)
A	1	No	58.99	27.19
A	2	Yes	71.02	5.20
B	1	No	84.06	24.73
B	2	Yes	93.57	35.22
C	1	No	90.15	3.56
C	2	Yes	31.34	30.15
D	1	No	56.25	6.26
D	2	Yes	66.64	7.51
E	1	No	53.71	40.66
E	2	Yes	72.25	33.54
F	1	No	33.51	31.14
F	2	Yes	57.94	32.69
G	1	No	66.23	16.34
G	2	Yes	70.84	8.81
H	1	No	34.28	11.75
H	2	Yes	33.04	12.46
I	1	No	47.67	15.93
I	2	Yes	51.58	15.35
J	1	No	67.78	23.01
J	2	Yes	71.54	13.80
K	1	No	40.94	11.22
K	2	Yes	38.92	9.94
L	1	No	83.44	17.49
L	2	Yes	76.18	10.40
M	1	No	45.55	11.25
M	2	Yes	53.98	7.90
N	1	No	38.60	4.59
N	2	Yes	37.28	7.13
O	1	No	42.28	3.34
O	2	Yes	47.72	0.83
P	1	No	47.41	5.06
P	2	Yes	51.01	4.32
Q	1	No	58.84	7.93
Q	2	Yes	65.80	2.68
R	1	No	32.84	3.68
S	1	No	29.18	14.69
S	2	Yes	23.28	12.76
T	1	No	38.69	3.58
T	2	Yes	31.22	19.94
U	1	No	32.38	2.54
U	2	Yes	30.60	11.41
V	1	No	19.42	14.01
V	2	Yes	27.33	14.78
W	1	No	11.64	6.26
W	2	Yes	12.46	6.12
X	1	No	28.82	0.98
X	2	Yes	25.68	3.34

Table S2. Mean and standard deviation of FWHM and 2nd Ratio for each trial.

ID	Trial	Selected	Mean FWHM (s)	SD FWHM (s)	Mean 2nd Ratio	SD 2nd Ratio
A	1	No	0.19	0.05	0.29	0.06
A	2	Yes	0.18	0.03	0.30	0.02
B	1	No	0.14	0.05	0.34	0.08
B	2	Yes	0.19	0.09	0.55	0.18
C	1	No	0.16	0.03	0.35	0.07
C	2	Yes	0.27	0.14	0.66	0.57
D	1	No	0.15	0.06	0.61	0.34
D	2	Yes	0.16	0.04	0.22	0.02
E	1	No	0.26	0.10	0.21	0.02
E	2	Yes	0.17	0.04	0.26	0.00
F	1	No	0.21	0.08	0.36	0.01
F	2	Yes	0.22	0.12	0.29	0.20
G	1	No	0.19	0.04	0.49	0.18
G	2	Yes	0.18	0.03	0.63	0.05
H	1	No	0.18	0.08	0.62	0.23
H	2	Yes	0.20	0.08	0.62	0.18
I	1	No	0.24	0.11	0.52	0.09
I	2	Yes	0.25	0.11	0.57	0.03
J	1	No	0.18	0.04	0.37	0.11
J	2	Yes	0.16	0.02	0.48	0.09
K	1	No	0.23	0.04	0.39	NaN
K	2	Yes	0.22	0.05	0.38	0.03
L	1	No	0.16	0.04	0.49	0.09
L	2	Yes	0.13	0.03	0.47	0.06
M	1	No	0.27	0.04	0.46	0.27
M	2	Yes	0.27	0.04	0.31	0.18
N	1	No	0.13	0.03	0.61	0.07
N	2	Yes	0.13	0.03	0.61	0.08
O	1	No	0.36	0.05	NaN	NaN
O	2	Yes	0.37	0.03	NaN	NaN
P	1	No	0.46	0.17	0.52	0.06
P	2	Yes	0.42	0.08	NaN	NaN
Q	1	No	0.25	0.00	0.20	0.05
Q	2	Yes	0.25	0.00	0.21	0.01
R	1	No	0.85	0.20	0.78	0.09
S	1	No	0.26	0.04	NaN	NaN
S	2	Yes	0.28	0.07	NaN	NaN
T	1	No	0.25	0.00	0.25	0.07
T	2	Yes	0.35	0.13	0.32	0.15
U	1	No	0.25	0.04	0.66	0.10
U	2	Yes	0.25	0.06	0.44	0.08
V	1	No	0.38	0.08	NaN	NaN
V	2	Yes	0.46	0.27	0.63	0.34
W	1	No	0.38	0.04	0.97	NaN
W	2	Yes	0.30	0.04	0.18	NaN
X	1	No	0.82	0.06	0.89	0.08
X	2	Yes	0.72	0.06	0.98	0.06

S2. Additional Figures

Figure S1 shows an example of a swing motion performed by an experienced practitioner.

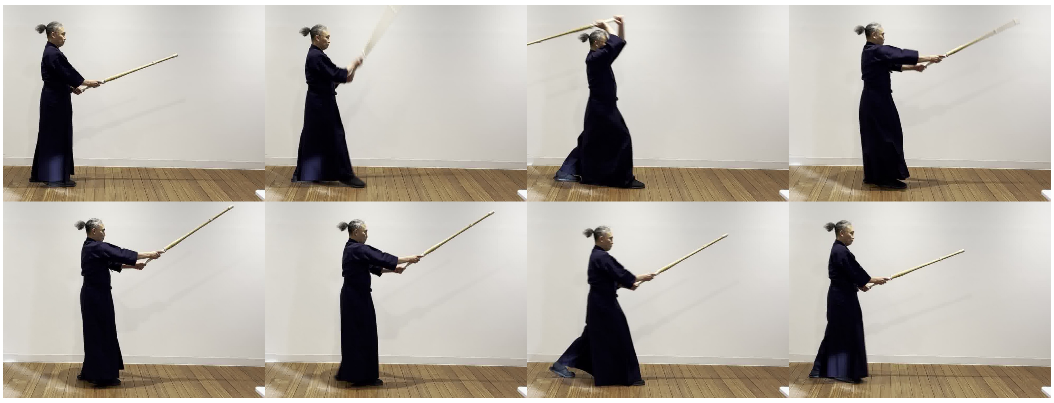

Figure S1. Example of a kendo swing motion performed by an experienced practitioner. Frames are extracted from the video every 0.33 s and displayed in a grid, with the first four frames in the top row and the subsequent frames in the bottom row.

Figure S2 shows the circuit diagram combining the Raspberry Pi Zero W and the MPU-9250.

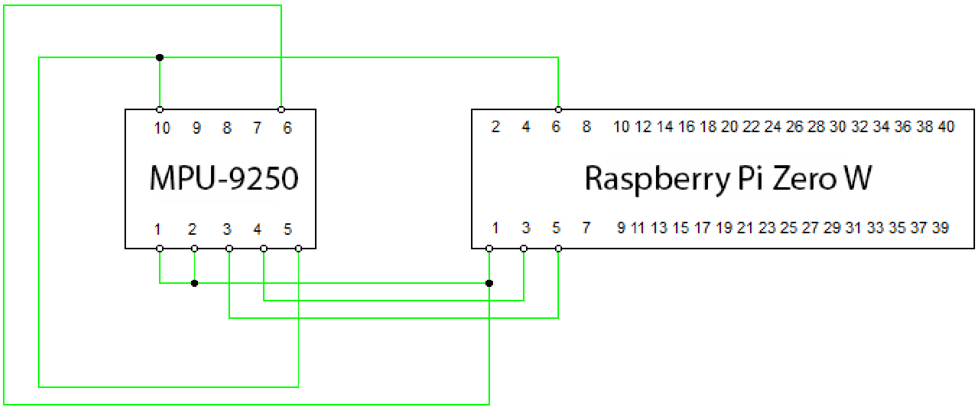

Figure S2. Circuit diagram combining the Raspberry Pi Zero W and the MPU-9250.

Figure S3 shows the time series of raw acceleration data measured from experienced kendo practitioner A during a swing trial. Figure S4 shows the time series of gyroscope data measured from the same trial.

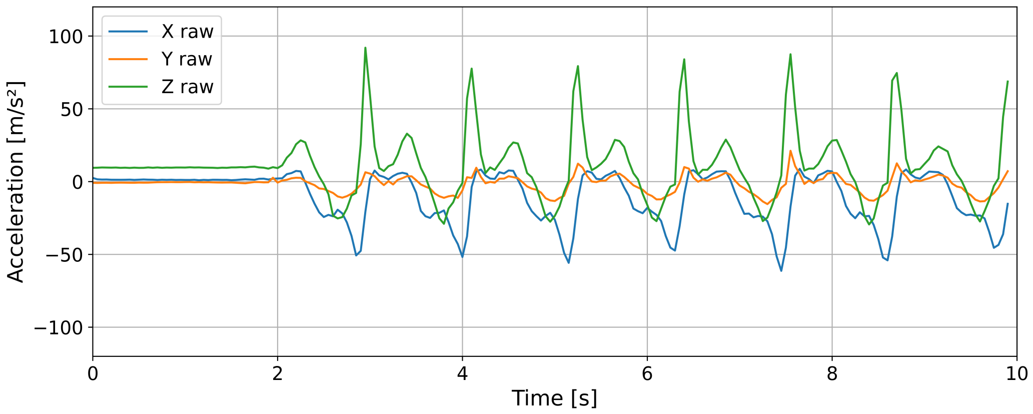

Figure S3. Time series of acceleration data measured from experienced kendo practitioner A during a swing trial.

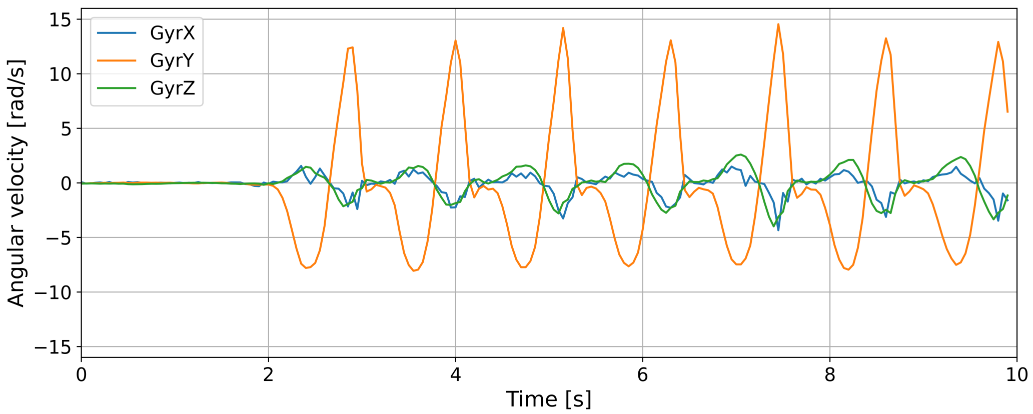

Figure S4. Time series of gyroscope data measured from experienced kendo practitioner A during a swing trial.
